# Investigation of Anti-fouling and UV-Cleaning Properties of PVDF/TiO_2_ Mixed-Matrix Membrane for Humic Acid Removal

**DOI:** 10.3390/membranes11010016

**Published:** 2020-12-24

**Authors:** Yeit Haan Teow, Boon Seng Ooi, Abdul Latif Ahmad, Jit Kang Lim

**Affiliations:** 1 Department of Chemical and Process Engineering, Faculty of Engineering and Built Environment, Universiti Kebangsaan Malaysia, Bangi 43600, Selangor Darul Ehsan, Malaysia; 2 Research Centre for Sustainable Process Technology (CESPRO), Faculty of Engineering and Built Environment, Universiti Kebangsaan Malaysia, Bangi 43600, Selangor Darul Ehsan, Malaysia; 3 School of Chemical Engineering, Engineering Campus, Universiti Sains Malaysia, Seri Ampangan, Nibong Tebal 14300, Penang, Malaysia; chobs@usm.my (B.S.O.); chlatif@usm.my (A.L.A.); chjitkangl@usm.my (J.K.L.)

**Keywords:** PVDF membrane, humic acid, TiO_2_ nanoparticles, fouling mitigation, UV cleaning

## Abstract

Natural organic matters (NOMs) have been found to be the major foulant in the application of ultrafiltration (UF) for treating surface water. Against this background, although hydrophilicity has been demonstrated to aid fouling mitigation, other parameters such as membrane surface morphology may contribute equally to improved fouling resistance. In this work, with humic acid solution as the model substance, the effects of titanium dioxides (TiO_2_) types (PC-20, P25, and X500) on membrane anti-fouling and defouling properties were comparatively analysed. The aims are (1) to determine the correlation between membrane surface morphology and membrane fouling and (2) to investigate the anti-fouling and UV-cleaning abilities of PVDF/TiO_2_ mixed-matrix membranes with different membrane topographies and surface energy conditions. The mixed-matrix membrane with P25 TiO_2_ exhibited the most significant UV-defouling ability, with a high irreversible flux recovery ratio (IFRR(UV)) of 16.56 after 6 h of UV irradiation, whereas that with X500 TiO_2_ exhibited both superior anti-fouling and defouling properties due to its smoother surface and its highly reactive surface layer.

## 1. Introduction

Progress has been made in using membrane technology to remove natural organic matters (NOMs) from water to comply with stringent water-quality regulations [1,2,3]. For the removal of humic acid (HA) from water, ultrafiltration has been shown to yield HA retentions ranging from 85 to 90% [4,5], whereas nanofiltration rejection could be more than 90% at pH 8 [6]. However, the chief operational obstacle to the wide application of membrane technology is the undesirable phenomenon of membrane fouling [7,8]. To address this, a periodic hydraulic backwashing is usually engaged; unfortunately, fouling is not usually always removed by backwash. Irreversible fouling happens when matters deposited both on the membrane surfaces and within the pores could not be removed, thus progressively compromising membrane performance [9,10].

Parameters such as membrane morphology, surface charge, and surface wettability have been established to crucially affect membrane fouling [11,12]. Nystrom et al. [13] investigated the fouling of HA on various filters and noticed that fouling was more severe for filters with larger pores. Lee et al. [14] investigated membrane fouling in microfiltration and ultrafiltration with four types of surface waters. They concluded that the shape and size of molecules and the membrane roughness affected the decline in flux and that rougher microfiltration membranes were more likely to be fouled than smoother ultrafiltration ones. The literature thus offers much evidence that the affinity between solutes and membranes, described by parameters such as sorption and aggregation of solutes on membrane surfaces and into the membrane pores, is the dominant factor governing fouling. Accordingly, approaches based on tailoring membrane surface properties have been suggested to minimize fouling [15,16]. Generally, hydrophilic membranes present improved anti-fouling and flux recovery properties. Kabsch-Korbutowicz et al. [17] studied the deterioration of permeation flux during ultrafiltration treatment of HA and noticed that hydrophilicity was a desirable property for the membrane surface that could mitigate fouling. This finding was corroborated by Lee at al. [18], who found that regenerated cellulose membranes mitigated fouling better than polyethersulfone membranes, mainly due to the former’s greater surface hydrophilicity. However, polymers used in membrane fabrication are mostly weakly hydrophilic [19] and therefore susceptible to fouling. Polyvinylidene fluoride (PVDF) is one example of a polymer which has outstanding thermal and mechanical strength [20] and has been extensively used in many applications. 

Studies have focused on incorporating additives into membrane matrix for the modification of polymeric PVDF membrane surfaces: these include inorganic additives (e.g., silica (Si), silica oxide (SiO_2_), zirconium dioxide (ZrO_2_), or titanium dioxide (TiO_2_) particles) [21,22,23,24] or hydrophilic polymers (e.g., cellulose acetate phthalate (CAP), polyvinyl pyrrolidone (PVP), or polyvinyl alcohol (PVA)) [25,26,27]. Nanotechnology-based advances have expanded avenues of membrane modification through the introduction of nanoparticles (NPs) in fabricating nanocomposite membranes. The incorporation of inorganic materials into organic polymer matrices has garnered much interest owing to the simplicity, mild conditions, and stable performance of the resulting blend. Beneficial effects of NPs-based membranes on mitigating fouling have copiously been reported, such as self-cleaning/anti-fouling properties [28,29]. This is mainly due to the hydrophilic enhancement of the membrane by incorporated NPs. However, the NP-related membrane functionality depends on the dispersibility of NPs in the membrane matrices.

The unique large surface-to-volume ratio and strong reactivity properties of TiO2 NPs with photocatalytic properties make them an ideal candidate to be incorporated into polymeric matrix as a hydrophilic filter. This research therefore aims to evaluate the performance of PVDF/TiO_2_ mixed-matrix membranes (MMMs) with respect to their long-term anti-fouling potential as a function of the dispersibility and photocatalytic activity. Three different commercial TiO_2_ NPs, P25, PC-20, and X500, with different crystallographic structures and particle distributions, were studied in the absence of dispersing aids. The resultant membrane properties were evaluated as follows: anti-fouling properties through relative flux reduction (RFR) parameters, defouling properties due to surface roughness through the flux recovery ratio (FRR), and irreversible fouling degradation through the irreversible FRR (UV) index.

## 2. Materials and Methods

### 2.1. Materials

PVDF (TA6010/1001, purchased from Solvay Solexis, Shanghai, China) was dissolved in N-N-dimethylacetamide (DMAc) (purchased from Merck, Darmstadt, Germany; with Assay GC area % ≥ 99%) and then cast at 200 μm thickness for the fabrication of PVDF/TiO_2_ MMMs. Three commercially available inorganic photocatalytic TiO_2_ nanopowders of different particle sizes were procured: <8 nm (X500) and 20 nm (PC-20) from TitanPE Technologies, Inc., Shanghai, China; 21nm (P25) from Sigma-Aldrich, St. Louis, MO, USA. According to the manufacturers’ specifications (see Table 1), PC-20 (20 nm) contains approximately 85% anatase and 15% rutile; P25 (21 nm) contains approximately 75% anatase and 25% rutile; and X500 (8 nm) is fully anatase. The nanoparticles (NPs) were used as received.

Synthetic humic acid (HA) with molecular weight in the range of 20,000–50,000 g/mol (obtained from Sigma-Aldrich, St. Louis, MO, USA) was used as the organic foulant during the experiment without further purification. The feed solution in the membrane filtration process was prepared by dissolving 10 mg of HA powder in 5 L of distilled water. The solution was neutralized to pH 7 via titration using 0.1 M sodium hydroxide, NaOH (supplied by Sigma Aldrich, St. Louis, MO, USA), with rigorous stirring for complete dissolution of HA in water. To study the effect of membrane fouling by HA, analytical grade anhydrous calcium chloride, CaCl_2_ (R & M Chemicals, Essex, U.K.), was added to the mentioned feed solution to adjust the total ionic strength.

### 2.2. Preparation of Stable TiO_2_ Colloidal Suspension

The TiO_2_ colloidal suspension to be used as a coagulation bath for the membrane phase inversion in the latter stages of membrane fabrication was stabilized via mechanical agitation (ultrasound) and by adjusting the pH value of the suspension to 4.0 in order to achieve electrostatic stability between TiO_2_ molecules [11]. At pH = 4.0, the zeta potential typically ranges between −30 mV and +30mV, meaning particles in suspension tend to repel each other, thus preventing agglomeration [4]. The suspension was subjected to ultrasonic irradiation for a 15-minute duration using a Telsonic ultrasonic horn SG-24-500P at 18.4 kHz (from Telsonic Ultrasonics, Inc., Macomb County, MI, USA) to further break apart the agglomerated TiO_2_ cluster. The colloidal suspension stability was determined by its particle size distribution (apparent hydrodynamic size) and the zeta potential of TiO_2_ nanoparticles with the use of a Malvern Zetasizer Nano NS90 (Malvern Instruments Ltd., Malvern, U.K.), employing the basis of dynamic light scattering (DLS) theory and cumulant method. The measurements were taken from a clear disposable zeta cell at 25 °C, where the TiO_2_ refractive index (RI) and absorption were set at 2.500 and 0.10, respectively [30,31]. Water (viscosity at 0.8872 cP and RI at 1.330) was chosen as the dispersant for the measurement. To minimize analytical error, the readings were taken from three measurements, each of fifteen runs for TiO_2_ particle size distribution and twenty runs for zeta potential, respectively.

### 2.3. Membrane Formation and In-situ Particle Embedment

Flat sheet PVDF/TiO2 MMMs were fabricated via an in-situ colloidal precipitation method, whereby nascent membrane polymer films were immersed into a stable TiO_2_ colloidal suspension [32]. Pre-dried PVDF (baked for 24 h in an oven at 70 °C to remove excess moisture) was dissolved into DMAc solvent in a beaker for the preparation of membrane casting solution. The composition of PVDF/DMAc was kept constant at a weight percentage ratio of 18:82.

The mixture was constantly stirred at 250 rpm, maintaining the temperature at 65 °C for 4 h until PVDF was completely dissolved, for the formation of homogenous membrane polymer solution. Afterwards, the temperature was reduced to 40 °C and the homogenous membrane polymer solution was left overnight with continuous stirring. Solvent evaporation was negligible considering the high boiling point of DMAc (164–166 °C).

The membrane polymer solution was cast using an automatic thin-film applicator (Elcometer 4340, Elcometer (Asia) Pte. Ltd., Singapore) on a flat glass plate wrapped with tight non-woven polyester fabric (Holleytex 3329, Ahlstrom, Helsinki, Finland) to form a membrane polymer film at a nominal thickness of 200 μm. The polyester fabric acts as a membrane support layer, providing mechanical strength to the membrane to withstand high pressure during operation. Once the membrane casting process was completed, the nascent membrane together with the glass plate were immediately removed from the platform and gently immersed into the coagulation bath (TiO_2_ colloidal suspension at 0.01 g/L concentration prepared in Section 2.2) for phase inversion. Such manner of incorporating nanoparticles into the membrane matrix is considered as an in-situ method, as membrane surface solidification and nanoparticle embedment occur simultaneously. The setup was then left immersed for a day to allow complete solidification and diffusion of residual solvent from the membrane. Finally, the fabricated membrane was lifted out of the coagulation bath after detaching it from the glass plate and rinsed with and then soaked in a bath of distilled water. Lastly, the fabricated membranes were each assigned denotation (see Table 2) as references for the following experiments.

### 2.4. Membrane Characterization

#### 2.4.1. Field Emission Scanning Electron Microscopy (FESEM) and Energy Dispersive X-ray (EDX) Analysis

The surface morphology of the synthesized PVDF/TiO_2_ MMMs was examined using a FESEM (SUPRA 35 VP, Carl Zeiss Inc. Oberkochen, Germany). The membranes were first briefly immersed in ethanol and then dried with filter paper to remove residue ethanol on the surface, before being air-dried. Membranes were then cut into small, appropriately-sized pieces of samples and were mounted onto the sample holders. A K 550 sputter coater was then used to coat the outer surfaces of the membrane samples with a thin conducting layer of gold (~20 nm thickness, 0.5 min elapsed) under vacuum to provide electrical conductivity and to prevent the surfaces from being electrically charged. Afterwards, the samples were examined under the electron microscope at 10.0 kV potential. The same membrane samples used in FESEM were also examined under an EDX (EDAX Inc., Mahwah, NJ, U.S.A.) at 500x magnification to capture the composition mapping and quality of TiO_2_ nanoparticles dispersion on membrane surfaces.

#### 2.4.2. Pore Size Distribution

Membrane pore size distribution was analysed with a Capillary Flow Porometer Porolux 1000 (Benelux Scientific, Ede, Netherlands) using the “dry up-wet up” method (round membrane samples of 10 mm in diameter). Using this method, gas flow was measured as a function of transmembrane pressure, by initially wetting the membrane samples with perfluoroether, followed by dry flow of gas through the membrane samples. The pore size distribution was then estimated using PMI software (Benelux Scientific, Ede, Netherlands).

#### 2.4.3. Atomic Force Microscopy (AFM)

The surface morphology and roughness of the membranes were analysed using an AFM XE-100 (Park Systems, USA). The measurements were taken at ambient conditions and the membranes were dried at room temperature prior to analysis. Membrane samples were cut into approximately 1 cm^2^ sizes and each sample was secured on the top of a scanner tube with carbon tape, followed by scanning with the laser beam reflected by the cantilever, within a scanning area of 10 µm × 10 µm. Generally, three measurements are conventionally used to define roughness: the mean roughness (*R_a_*), the root mean square roughness (*R_q_*), and the average difference in the heights between the highest and the lowest points (*R_z_*). In this study, *R_q_* was used as the evaluation measurement to compare the roughness of the membranes produced and was the defined roughness parameter studied using the AFM XE Data Acquisition program in non-contact mode. Mathematically, *R_q_* is defined as the average value of the surface relative to the central plane for which the volumes enclosed by the images above and below the plane were equal, as represented in Equation (1):

(1) $$R_{q}={\left[\frac{1}{S}\int_{0}^{a}\int_{0}^{b}{\left\{f(x,y)-z_{m}\right\}}^{2}dxdy\right]}^{\frac{1}{2}}$$

where *f*(*x*,*y*) is the height in the specified area, *S* is the specified area, *a* and *b* are the length of the sample, and *Z_m_* is the mean height value.

#### 2.4.4. Surface Tension (Wettability)

Two different approaches were used to characterize the membranes’ surface tension or wettability, namely, automated goniometer, and probe liquids.

*Automated goniometer*: The membrane surface wettability was characterized by the static contact angle of the membrane samples with a liquid, which was measured based on the sessile drop technique using a DropMeter A-100 contact angle system (Rame-Hart Instrument Co., Succasunna, NJ, U.S.A.). Dry membrane samples were adhered onto a glass slide using double-sided tape to flatten the surface and to orientate the test surface facing upwards. A droplet (~13 µL) of deionized water was then dropped onto the dry membrane surface using a motor-driven micro-syringe at room temperature. Following that, a microscope with a long working distance of 6.5× objectives was used to capture micrographs at high frequency (100 pcs/s). The acquired images were analyzed using DROPimage software to obtain the values of the contact angles. To minimize analytical error, the reported contact angles were averages of measurements taken at 10 different locations on each membrane surface sample.

*Probe liquids*: The surface free energy of the membranes was derived based on an acid-based (van Oss) approach that underpins the calculations. In this method, the contact angle was measured against three probe liquids with known physical properties; then, the measurements were substituted into a set of three first-order linear equations to calculate the membrane’s surface free energy. The contact angle probe liquids selected for this investigation were deionized water, glycerol, and formamide. Deionized water was dispensed from a TKA Smart2pure (Thermo Electron LED GmbH, Langenselbold, Germany) water purification system; glycerol (ultrapure, molecular biology grade with assay: ≥ 99.5%) was obtained from USB Corporation, USA; and formamide (A.C.S. reagent, 99.5+%) was procured from Sigma Aldrich, Malaysia. The physical properties of the probe liquids, namely, the Lifshitz-van der Waals component (*γ^LW^*), electron-acceptor component (*γ^+^*), electron-donor component (*γ^−^*), polar energy component (*γ^AB^*), total free energy component (*γ^TOT^*), and the viscosity (*η*) are tabulated in Table 3.

#### 2.4.5. X-Ray Diffraction (XRD)

XRD was used to examine the crystal structure of TiO_2_ nanoparticles and the crystalline phases of PVDF on the membrane surface. TiO_2_ nanopowder and membrane samples were subjected to X-ray radiation, and the diffraction data were collected on a diffractometer (D8 ADVANCE, Bruker AXS, GmbH). The system was equipped with a Cu X-ray tube (18 kW Cu Kα radiation; λ = 0.15418 nm) and a LynxEye detector operating at 60 kV and 80 mA, and the configuration was calibrated using a lanthanum boride (LaB6) powder standard (ICDD PDF#34-0427). Readings were taken at a scanning angle from 10° to 90° (2θ-angle), with a step width angle of 0.02^◦^ and a sampling time of 0.3 s per step width.

### 2.5. Permeation Flux, Anti-Fouling, Defouling, and UV-Cleaning Experiments

**Figure 1:**
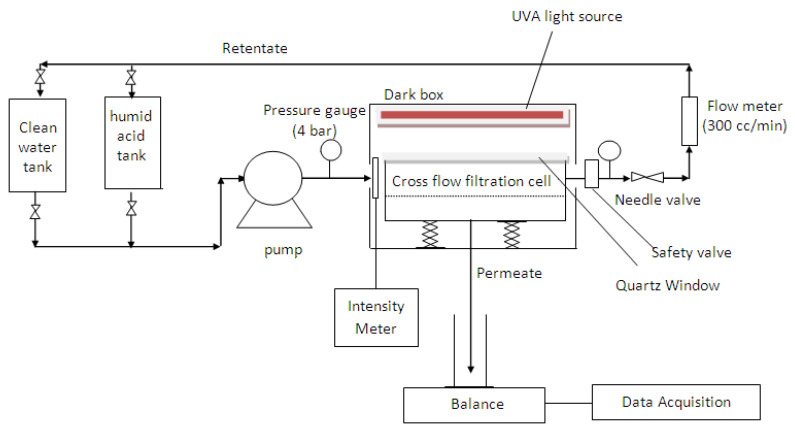
Schematic diagram of the laboratory bench scale cross-flow recirculation unit.

A laboratory bench scale cross-flow recirculation unit was used to study the permeation flux, anti-fouling, defouling, and UV-cleaning properties of the membranes. The schematic of the set-up is displayed in Figure 1, which consists of a membrane cross-flow filtration cell, two feed reservoirs (HA tank and clean water tank), a peristaltic pump (Hydra-Cell, Wanner International, U.K.), a flow meter, a balance connected with a data acquisition system to interpret the filtrate flow rate, a pressure gauge measuring the equilibrium pressure exerted on the filtration cell, a Philips UV-A light (Actinic BL TL-K 40 W/10-R) emitting near-UV radiation at long wave UV-A (350–400 nm), and an UV intensity meter (Sglux, sensor monitor 5.0). Within the crossflow filtration cell, each synthesized flat sheet membrane was cut and laid on top of the membrane holder in a custom-designed transparent rectangular membrane test cell with a size of 7.5 cm × 9.6 cm (effective membrane filtration area of 72.0 cm^2^, excluding the area covered by the O-ring) and was then tightened using a rubber O-ring.

The experiment was conducted in five successive steps but each under different experimental conditions. During the filtration process, fresh membranes can be compacted by the applied pressure, resulting in flux decline. To compensate for the effects of compaction, the experiment was first carried out with pure distilled water to obtain steady-state flux readings at a constant pressure of 0.5 bar for 4 h. To elaborate, fresh distilled water was charged into the 5 L feed reservoir (clean water tank) and re-circulated at a constant cross-flow rate of 0.04 L/min using a peristaltic pump. A constant pressure across the filtration unit was regulated at 0.5 bar using the needle valve, with the permeate side opened to the atmosphere. Permeation flux was then determined via direct measurement of the permeate volume over the permeation time,

(2) $$J=\frac{V}{At}$$

where *J* is the permeation flux (L/m^2^ h), *V* is the permeate volume (L), *A* is the membrane effective surface area (m^2^), and *t* is the permeation time (h).

The fouling experiment was performed for 12 h using 2 mg/L HA as model solution to simulate the organic matters that exist in fresh water. The pH of the HA solution was set at pH = 7.1, and mM CaCl_2_ was added to the HA solution in the 5L feed reservoir (HA tank) to provide cation strength, promoting HA fouling. During the filtration runs, the retentate was returned to the feed reservoir (HA tank) to minimize dilution effects. Additionally, fresh HA solution was added at 2-hour intervals to maintain a constant concentration. In order to evaluate the fouling resistance of a membrane, the RFR percentage, which is used to represent the fouling tendency of a membrane, was calculated as below:

(3) $$RFR(\%)=\left(1-\frac{J_{P}}{J_{W1}}\right)\times 100\%$$

where *J_P_* is the solution permeate flux (L/m^2^ h) and *J_W_*_1_ is the initial pure distilled water flux (L/m^2^ h). Generally, the lower the RFR value, the better the membrane’s anti-fouling properties. In such calculations, the fouling effect is quantified and derived from the measured resistance to flux caused by pore blocking or the formation of a gel layer on the membrane surface.

Once the HA solution had been continuously filtered for 12 h, hydraulic cleaning was then conducted in the same cross-flow manner using the distilled water from the feed reservoir for another 4 h to determine the flux recovery of the membrane. The measured flux recovery can in turn be correlated back to the defouling properties of a membrane, which is represented in the formula below (applicable after hydraulic cleaning):

(4) $$FRR(\%)=\left(\frac{J_{W2}}{J_{W1}}\right)\times 100\%$$

where *J_W_*_2_ is the pure water flux after washing (L/m^2^ h) and *J_W_*_1_ is the initial pure distilled water flux (L/m^2^ h). Generally, higher FRR (approaching unity) indicates better defouling properties of a membrane.

Subsequently, photocatalytic degradation of HA remnants on membranes was carried out by directly illuminating each fouled membrane with a 40 W UVA lamp (Actinic BL TL/TL-D/T5, Philips, Germany) with a light intensity of 1.53 mW/cm^2^ at the membrane surface for 3 h without water flow to activate the self-cleaning properties of embedded TiO_2_ nanoparticles. Afterwards, a water permeability test was conducted for 4 h to measure the transport characteristics of the membranes. The same process was repeated twice to plot out the relationship between the UV irradiation times with regards to the degree of HA degradation.

Finally, to quantify the efficacy of IFRR(UV) caused by HA photocatalytic degradation (after 6 h of UV illumination), compared to the control case (neat PVDF membrane), the ratio of water permeability after UV irradiation to that before irradiation was calculated as follows:

(5) $$IFRR(UV)(\%)=\left(\frac{J_{W3}-J_{W2}}{J_{W2}}\right)\times 100\%$$

where *J_W_*_3_ is the recovery water flux after irradiation with 6 h of UV light (L/m^2^ h) and *J_W_*_2_ is the pure water flux after washing with distilled water (L/m^2^ h) without UV light.

## 3. Results and Discussion

### 3.1. Morphologies of PVDF/TiO_2_ Mixed-Matrix Membrane

**Figure 2:**
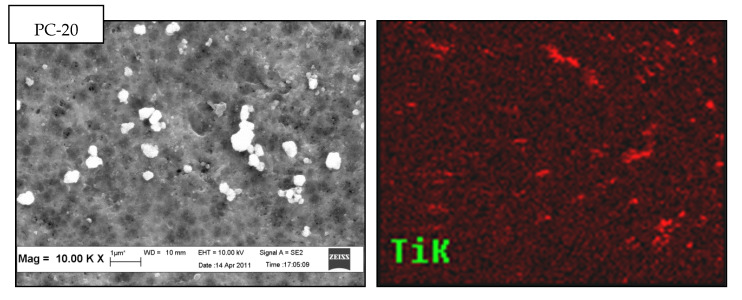

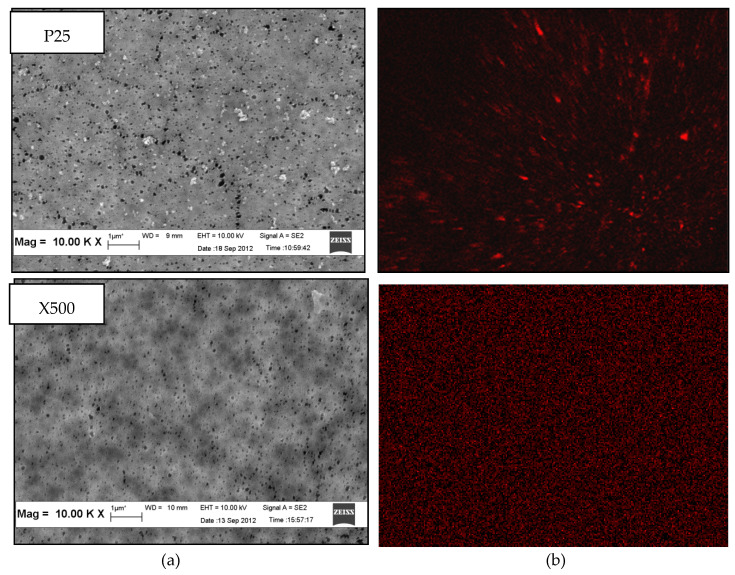
Top surface (**a**) field emission scanning electron microscopy (FESEM) micrographs and (**b**) energy dispersive X-ray (EDX) mapping of PVDF/TiO_2_ mixed-matrix membranes (MMMs) incorporated with different types of TiO_2_ (coagulation bath concentration = 0.01 g/L, pH = 4, ambient conditions).

Top surface FESEM micrographs of PVDF/TiO_2_ MMMs synthesized using DMAc as solvent and immersed in 0.01 g/L colloidal suspensions of different TiO_2_ nanoparticles types are shown in Figure 2. At 10.00 k× magnification (see Figure 2a), connected pores were observed on the surface of all membranes. This suggests that the choice of TiO_2_ nanoparticles does not contribute significantly to the structural change of membrane morphology. Note that contrary to PC-20 and P25 samples, TiO_2_ nanoparticles are not visible on the membrane surface image of PVDF/TiO_2_ MMMs synthesized using X500 TiO_2_ nanoparticles. However, the absence of visibility is hypothesized to be due to the nanoparticle size in X500 (average < 8nm) being too small to be observed by the FESEM at 10.00 k× magnification.

The presence and dispersion of TiO_2_ nanoparticles in the membrane structure was further confirmed by EDX mapping. The variation in distribution patterns of TiO_2_ nanoparticles in PVDF/TiO_2_ MMMs membrane surface structure was distinctly shown (in red colour) in Figure 2b. Obviously, X500 having the smallest TiO_2_ hydrodynamic cluster size in suspension was better dispersed into the membrane matrix compared to those containing PC-20 and P25 TiO_2_ nanoparticles. The lesser degree of TiO_2_ clustering in the membrane synthesized using X500 is attributed to its higher thermodynamic stability in the coagulation bath when the solvent and polymer were brought into contact. The observed TiO_2_ nanoparticle dispersion in the synthesized membranes is consistent with the theory proposed by Mackay et al. [34], whereby the thermodynamic stability of nanoparticles in a polymeric liquid is positively correlated with the ratio of the radius of gyration (*R_g_*) of the linear polymer to the radius of the nanoparticles (*R_p_*). Accordingly, the *R_g_/R_p_* ratio for PC-20, P25, and X500 was calculated to be 0.399, 0.921, and 4.846, respectively, with reference to the method proposed by Inagaki et al. [35]. With higher thermodynamic stability (represented by the *R_g_/R_p_* ratio), and also a comparatively smaller hydrodynamic cluster size (5–12 times smaller than PC-20 and P25), the agglomeration tendency of X500 nanoparticles in the colloidal solution was diminished and lead to more even particle size distribution during phase inversion with in-situ nanoparticle embedment. This outcome is consistent with Bagchi [36] who reported clustering is inhibited when particles exhibit robust stabilization, such as repelling each other sterically, which leads to better thermodynamic stability.

### 3.2. Pore Size Distribution

**Figure 3:**
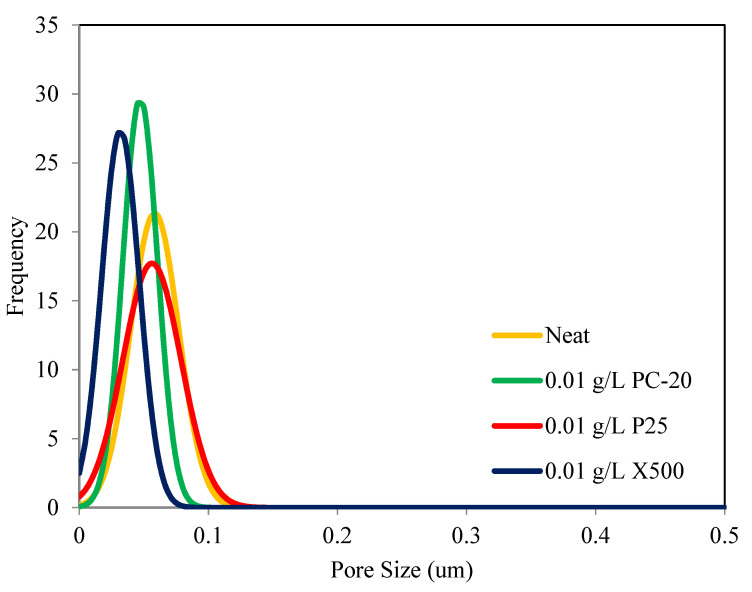
Pore size distribution of PVDF/TiO_2_ MMMs and neat PVDF membrane as a function of TiO_2_ type.

In-situ colloidal precipitation with a colloidal stable TiO_2_ suspension is an ideal method to synthesize PVDF/TiO_2_ MMMs with minimum changes to its physical properties. The pore size distributions of neat PVDF membranes and PVDF/TiO_2_ MMMs are shown in Figure 3. The mean pore diameter, *d_p,mean_*, of the neat PVDF membrane is approximately 0.059 μm, while for the membranes synthesized using PC-20, P25, and X500, TiO_2_ nanoparticles are measured at 0.047 µm, 0.058 µm, and 0.032 µm, respectively. Based on the distribution patterns across different membrane samples, it can be seen that the embedment of TiO_2_ nanoparticles had a more prominent effect on pore size alteration than pore size distribution. In other words, smaller TiO_2_ nanoparticle size impacted to a greater degree on membrane pore size shift. The distribution patterns also showed that embedment of PC-20 and P25 TiO_2_ nanoparticles did not cause significant change in membrane pore size, unlike the case for X500 displaying a pore-narrowing tendency when introduced into the membrane’s polymeric matrix. This is likely due to pore blocking when the TiO_2_ nanoparticles used are relatively smaller than the membrane pores and disagreed with the finding obtained by Wang et al. [37]. PVDF-PVP-TiO_2_ membrane synthesized by Wang et al. (2016) had a larger pore size with the addition of P25 TiO_2_ NPs [37]. Regardless, the pore size narrowing seen in X500 membrane samples is considered not significant. Overall, the results indicated that in-situ colloidal precipitation with a hydrophilic filler such as TiO_2_, in a stable colloidal suspension, is an ideal method to synthesize PVDF/TiO_2_ MMMs with minimum changes to their physical properties compared to PVDF TiO_2_ membranes synthesized by other researchers [38].

### 3.3. Atomic Force Microscopy (AFM)

**Figure 4:**
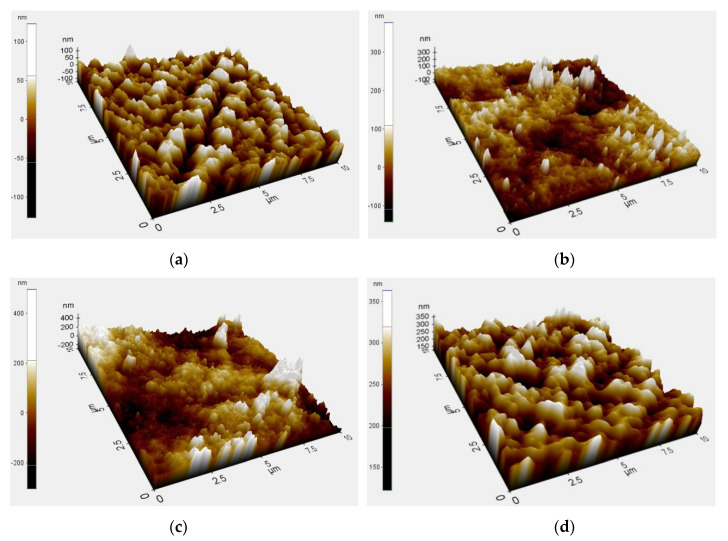
Atomic force microscopy (AFM) three-dimensional surface micrographs of (**a**) neat PVDF membrane and PVDF membrane immersed in a coagulation bath with different types of TiO_2_ at a concentration of 0.01 g/L, (**b**) PC-20, (**c**) P25, (**d**) X500.

Figure 4 shows the three-dimensional AFM micrographs of neat PVDF membrane and the synthesized PVDF/TiO_2_ MMM surfaces. The brightest area represents the highest point on the membrane surface, and the dark regions indicate the membrane pores. These images show that the membrane surfaces, especially those containing PC-20 and P25 TiO_2_ nanoparticles, were rough as the surface morphology was extensively altered by the clustering effect of TiO_2_ nanoparticles deposited within the PVDF membrane matrix. In addition, the ridge-and-valley structure on the MMMs is dissimilar, where uneven nodule aggregates can be seen at the surfaces of the PC-20 MMM and P25 MMM. The membrane surface roughness, expressed in terms of the root mean square of the Z data (*R_q_*) and the surface area ratio (%), were calculated using the XE Image Processing Program (Version 1.7.6), yielding the results in Table 4. Note that the surface area ratio was calculated by dividing the total surface area of the PVDF-TiO_2_ MMM (factoring in the ridge-and-valley structure on the membrane surface), with the flat surface of the calculated area in the denominator. The higher surface area ratio means that there are more ridge-and-valley structures present across the calculated surface area and therefore higher surface roughness. It can be seen in Table 4 that the TiO_2_ nanoparticles-embedded MMMs are of higher roughness than the neat PVDF membrane, with the exception of membrane synthesized with X500. For the PVDF/TiO_2_ MMMs synthesized with PC20 and P25 TiO_2_ nanoparticles, the larger particle size and more prominent clustering effect of TiO_2_ nanoparticles assembled on PVDF membrane surface created both deeper depressions (pores) and higher peaks (TiO_2_ nodules). This finding is contrary to the work conducted by Oh et al., probably due to difference in membrane synthesis method [39]. On the other hand, an improved *R_q_* value from X500 MMM compared to PC-20 MMM and P25 MMM is an indication of a uniform (hierarchical) surface, attributable to even dispersion of X500 nanoparticles into the membrane matrix. Note that membrane with a perfect hierarchical structure will give an *R_q_* value of zero.

### 3.4. Contact Angle

The contact angles of the neat PVDF membrane and PVDF/TiO_2_ MMMs synthesized with PC-20, P25, and X500 TiO_2_ nanoparticles are included in Table 5, whereby the contact angle of the neat PVDF membrane is the lowest, while the same value varies between different types of synthesized membranes. Theoretically, the contact angle is a function of membrane surface wettability, porosity, pore size, surface roughness, and pore size distribution. In general, higher surface roughness is expected to yield a greater contact angle when comparing between membranes with similar hydrophilicity [40]. Contact between water droplets and a rough membrane surface is impaired by the presence of microscopic air gap(s) between the polymer surface and water, thus lowering the membrane surface’s wettability.

In this study, the contact angles of the PVDF/TiO_2_ MMMs increase in the order of PC-20 < X500 < P25. Where PC-20 samples displayed a lower contact angle compared to P25 and X500 samples. The differences in these results is attributable to the underlying dispersion or aggregation of the TiO_2_ nanoparticles during membrane synthesis, as well as the crystalline phase composition of the TiO_2_ used. The lower contact angle seen on PC-20 (85% anatase; 15% rutile) compared to P25 (75% anatase; 25% rutile) is consistent with reported findings in the literature; whereby a greater ratio of anatase to rutile crystallinity correlates to higher hydrophilicity [41]. However, X500 samples which contain only anatase (100%) crystalline of TiO_2_ have a counterintuitively higher contact angle compared to PC-20 samples. This behaviour is attributed to both the rice-like crystalline structure of X500 TiO_2_ and the improved dispersion, which gave rise to a prominent hierarchical membrane surface structure (seen in Figure 4d). This kind of membrane structure is thought to induce the lotus effect, in which air gap(s) trapped between TiO_2_ nodules contributed to the reduction of membrane surface wettability (or hydrophilicity), hence the observed higher surface angle on X500 samples compared to PC-20.

To exclude the effect of surface roughness on membrane-water affinity, an extended Derjaguin-Landau-Verwey-Overbeek, DLVO (XDLVO) calculation proposed by van Oss [42] was applied to isolate and characterize the total interfacial tension and the free energy of interaction between the membrane surface and water molecules (*γ_sw_*), represented as

(6) $$\gamma_{sw}={\left(\sqrt{\gamma_{s}^{LW}}+\sqrt{\gamma_{w}^{LW}}\right)}^{2}+2(\sqrt{\gamma_{s}^{-}\gamma_{s}^{+}}+\sqrt{\gamma_{w}^{+}\gamma_{w}^{-}}-\sqrt{\gamma_{s}^{+}\gamma_{w}^{-}}-\sqrt{\gamma_{s}^{-}\gamma_{w}^{+}})$$

where *γ^LW^* is the Lifshitz-van der Waals component, and *γ^+^* and *γ^-^* are the electron-acceptor and electron-donor components respectively. The subscripts *s* and *w* each denote the solid surface (membrane) and the water molecule respectively.

Application of the XDLVO approach in Equation (6) requires that the terms representing the surface energy of the membrane (*γ_s_^LW^*, *γ_s_^+^*, and *γ_s_^−^*) be determined experimentally. However, using three different probe liquids, which came completely characterized from the manufacturer, together with the measured contact angles on the membranes, the surface tension components for Equation (6) can be calculated according to the extended Young equation, comprising both the apolar and polar interactions:

(7) $$\left(1+\cos\theta )\gamma_{l}^{TOT}=2(\sqrt{\gamma_{s}^{LW}\gamma_{l}^{LW}}+\sqrt{\gamma_{s}^{+}\gamma_{l}^{-}}+\sqrt{\gamma_{s}^{-}\gamma_{l}^{-}}\right)$$

where *θ* is the contact angle, and *γ^TOT^* is the total surface tension. The subscripts *s* and *l* each denote respectively the solid surface (membrane) and the liquid used in the contact angle measurement.

The total interfacial tension calculated using the XDLVO approach can then be used to evaluate the free energy of interaction between the membrane and water molecules, Δ*G_sw_*, following the equation proposed by Dupré [43]:

(8) $$\Delta G_{sw}=-2\gamma_{sw}$$

The free energy of interaction is the tendency of a liquid to disperse when it comes into contact with a solid surface. The value provides quantitative evaluation of the hydrophobicity or hydrophilicity of a membrane surface, while discounting the effects of surface roughness: *γ_sw_* < 0 or Δ*G_sw_* > 0 denotes a repulsion between water molecules and the membrane surface, which characterize the behaviour of a hydrophobic surface, whereas the reverse is true for a hydrophilic surface (*γ_sw_* > 0 or Δ*G_sw_* < 0). All of the calculated properties (i.e., surface energy of membrane, total interfacial tension, and free energy of interaction between membrane surface and water molecules) of the neat PVDF membrane and of the PVDF/TiO_2_ MMMs synthesized using PC-20, P25, and X500 as hydrophilic filler are summarized in Table 5. Based on the results, P25 and X500 MMMs are more hydrophilic than the neat PVDF membrane, while the PC-20 MMM displayed the most hydrophobic (or least hydrophilic) properties due to a higher degree of PC-20 TiO_2_ nanoparticles clustering on the membrane surface, which in turn inhibits the wettability of the surface. Hence, the membranes’ hydrophilicity decreases in the order of X500 > P25 > Neat > PC-20. Note that these calculations contradict the trend observed (Neat > PC-20 > X500 > P25) when the hydrophilicity is measured by the contact angles with water as the probe liquid. This implies that when hydrophilic nanoparticles are dispersed into a polymeric matrix, the surface roughness plays a dominant role in enhancing the functionality of the membrane. In order to improve the membrane surface wettability, the nanoparticles must be finely dispersed to facilitate the formation of a hydrated layer at the membrane surface.

### 3.5. XRD

**Figure 5:**
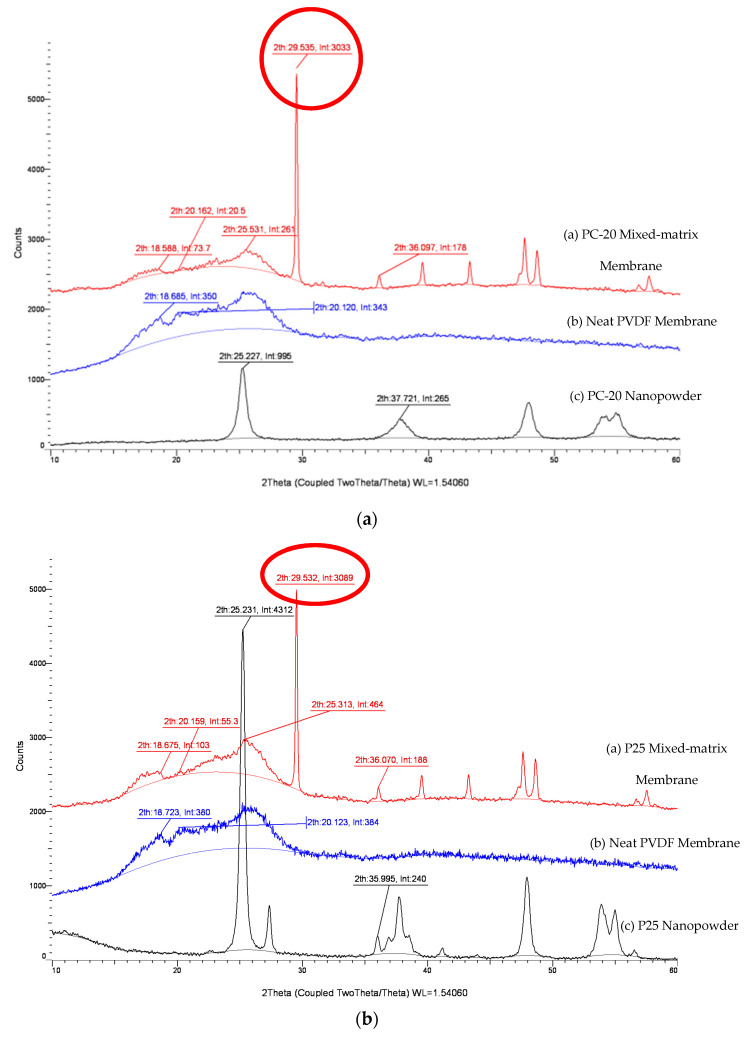

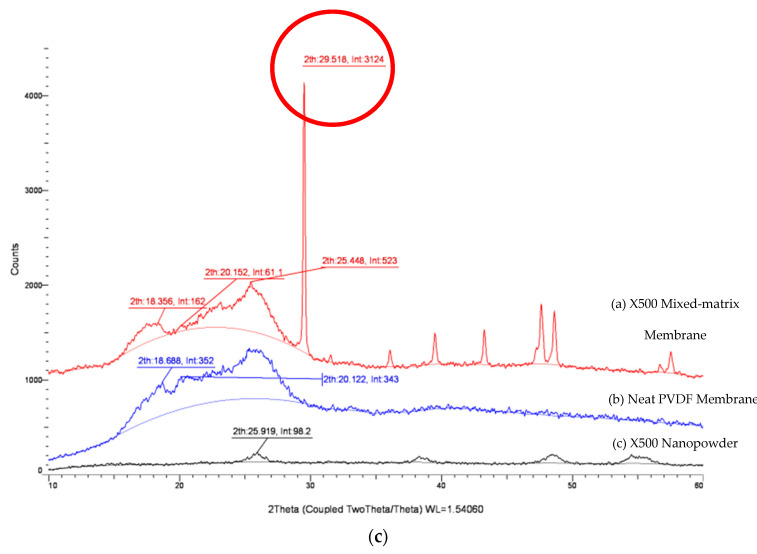
XRD patterns of PVDF/TiO_2_ MMMs, neat PVDF membrane and TiO_2_ nanopowder. (**a**) PC-20, (**b**) P25, (**c**) X500.

Changes to a membrane’s hydrophilicity/hydrophobicity properties from addition of inorganic nanoparticles is a complicated phenomenon. Evidence indicates that not only the Lewis and acid-base interactions between the membrane surface and the solution is altered, but the embedment of fine particles with different sizes and crystallinity also induced polymorphic changes to the PVDF matrix. Figure 5 shows the XRD crystalline peaks for the MMMs with different types of TiO_2_ incorporated. By integrating the area under the crystalline peaks, the XRD pattern of P25 TiO_2_ membrane is shown to contain an additional rutile phase (2θ of 27.6° and 35.995°) compared to PC-20 and X500. As discussed in Section 3.4, a TiO_2_-incorporated composite membrane with a higher proportion of anatase crystalline phase should exhibit more tendencies of hydrophilic behaviours. However, this statement seems to contradict the free energy analysis, whereby PC-20 (with more anatase) was found to have decreased water affinity (less hydrophilic) compared to P25 (with less anatase). This phenomenon is inferred to be due to induced polymorphism change of PVDF, consistent with the reports in the literature whereby PVDF with a higher α-polymorph displays more hydrophilic properties than expected [44].

The results in Figure 5 reveal that a prominent and distinct polymorphic change is observed after the inclusion of TiO_2_ nanoparticles in the neat PVDF membrane. The crystal planes associated with the characteristics of an α-polymorph of the PVDF membrane at 29.5°, which was close to the literature value of 27.6° from Dillon et al. [45] and Buonomenna et al. [46], deviated higher with the addition of TiO_2_ nanoparticles. The intensities of the α-polymorph peaks varied among the TiO_2_ MMMs, with the lowest value observed for PC-20. This reading is consistent with the calculations done earlier whereby PVDF-PC-20 has the lowest attractive free energy among the mixed-matrix membranes (MMMs).

### 3.6. Membrane Anti-Fouling Properties and UV-Cleaning Potentials

#### 3.6.1. Water Permeation Test

For the water permeation test, the flux data are presented in terms of normalized flux, represented by the instantaneous flux over the initial flux (*J/J_O_*). The effect of TiO_2_ nanoparticles type (PC-20, P25, X500) on the performance of MMMs was studied at a TiO_2_ concentration of 0.01 g/L, while the CaCl_2_ concentration and pH value were adjusted to 1 mM and pH = 7.0, respectively.

The initial water flux values for all the TiO_2_ embedded membranes are tabulated in Table 6. Note that for each type of membrane, the values reported were an average of three replications on independent sample membranes that were chosen randomly. The permeate fluxes indicate that the MMMs are relatively more permeable with the addition of TiO_2_; this is postulated to be due to (1) increase in hydroxyl groups on the TiO_2_ surface enhancing the permeate water flux [12] and (2) increase in membrane structure porosity, as substantiated by the water permeate flux performance of PC-20 MMM, despite it displaying more hydrophobic properties.

Accordingly, PVDF/TiO_2_ MMMs with homogeneous distribution of X500 nanoparticles displayed the best membrane water permeability, suggesting that X500 (fully anatase) possesses the most hydrophilic behaviour compared to the other membranes, and would be best suited to mitigate the fouling effects when operating with HA solution. This mitigation is attributed to higher permeability towards water molecules due to increased adsorption sites (a function of the nanoparticles’ surface areas) towards water molecules, which is promoted by the homogenous dispersion of X500 nanoparticles [47].

#### 3.6.2. Anti-Fouling Properties of The Membranes (RFR)

Considering that groundwater typically has a hardness level above 300 mg/L of dissolved calcium and magnesium [48], calcium was added to the HA feed solution to explore its effects on membrane fouling behaviour. The divalent Ca^2+^ cation in the HA solution binds with the negatively charged carboxyl functional groups on the HA molecules and effectively reduced the electrostatic repulsion amongst HA molecules, as well as between the HA molecules and the negatively charged membrane surface. This binding effect between Ca^2+^ cations and carboxyl groups causes HA molecules to reaggregate into coiled and spherical mass [49], as evidenced in findings reported by Yuan and Zdyney [50], whereby the portion of HA with molecular weight greater than 300 kD in a solution rose from only 1% to 9% in the presence of 1 mM CaCl_2_. When the electrical repulsion between HA molecules and the negatively charged membrane surface is decreased, adsorption onto the membrane sets in as a result, and the adsorbed layer subsequently induces a sharp drop in permeate flux within minutes of filtration operations.

**Figure 6:**
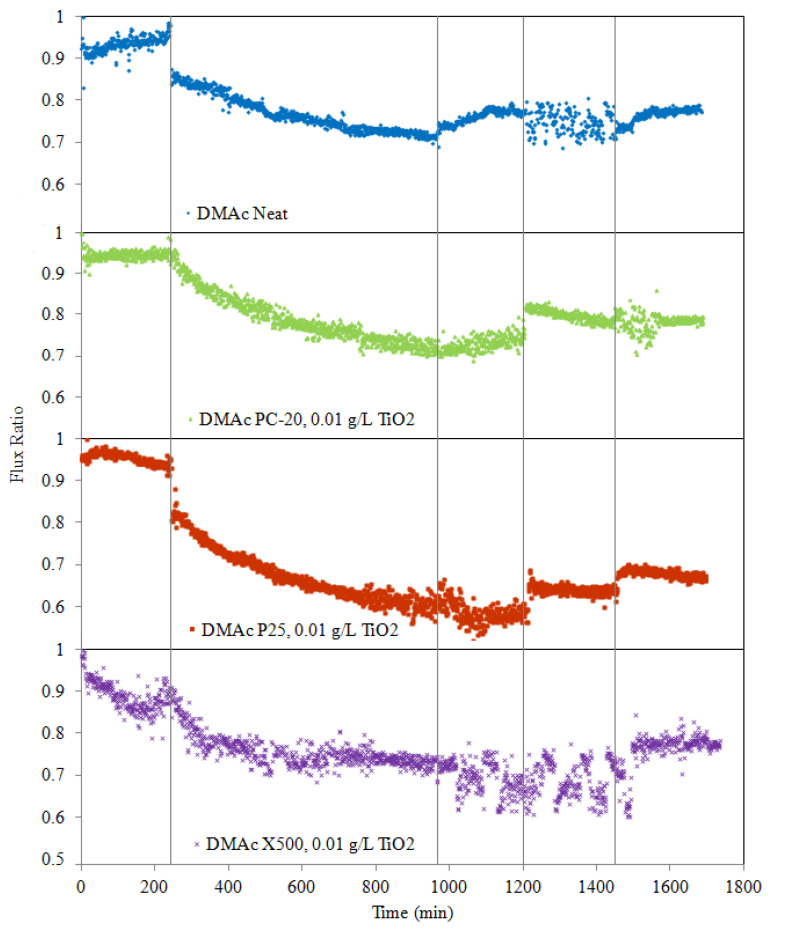
Effect of TiO_2_ type on PVDF/TiO_2_ MMM anti-fouling potential during five stages: pure water flux for 4 h, HA solution filtration for 12 h, hydraulic cleaning pure water flux for 4 h, water flux for 4 h after 3 h of UV irradiation, and water flux for 4 h after a 2nd round of 3 h of UV irradiation. Operation conditions during the experiment: [HA] = 2 mg/L; [Ca^2+^] = 1 mM (as CaCl_2_); pH 7.0; temperature = 25 ± 1 °C; cross-flow velocity = 4.0 L/hr, operating pressure = 0.5 bar; light intensity = 1.53 mW/cm^2^.

The permeate flux data collected from successive filtration cycles of the membranes are presented in Figure 6. Note that the HA fouling tendency for each of the membranes, represented by the RFR values, are 24.24% (neat PVDF), 23.48% (PC-20), 36.07% (P25), and 14.69% (X500), after 12 h of HA filtration (the end of HA fouling). As the membrane pore size for PC-20, P25, and X500 MMMs used in this study were much smaller than the equivalent size of HA aggregates, conventional wisdom might suggest the membrane resistance would be similar between each other membrane. However, the results do reveal a correlation between the pore size distribution and the membrane fouling resistance for X500 and PC-20 MMMs, which have smaller pore sizes compared to the neat membrane and P25 MMM, and they were found to have more fouling resistance in comparison. Subsequent accumulation on the membrane surface eventually resulted in a diminishing effective membrane pore size, thus leading to further physical retention and consequently increase in hydraulic resistance.

Membrane fouling phenomenon could be influenced by hydrodynamic conditions such as permeation drag and back transport, as well as chemical interaction between foulants and membranes [51]. To minimize the effects of those mentioned variables, the membranes were tested under similar hydrodynamic conditions and with similar membrane physical properties. In such manner, the relationship between membrane hydrophilicity and anti-fouling properties can be isolated in this study. Furthermore, as this paper has shown thus far, addition of TiO_2_ imparts changes to membrane properties (one of it being hydrophilicity), and by extension, fouling behaviours can then be correlated back to the conditions pertaining to TiO_2_ entrapment into membranes.

Theoretically, the incorporation of hydrophilic TiO_2_ nanoparticles into the PVDF membrane matrix could reduce the membrane’s fouling propensity due to higher affinity of TiO_2_ towards water (increased hydrophilicity). The underlying mechanism whereby the anti-fouling behaviour induced by TiO_2_ nanoparticles is known in the literature to be due to water shielding caused by the hydrophilic –OH groups on the membrane surface [51]. However, data from Figure 6 indicates that although hydrophilicity was improved with the incorporation of TiO_2_, pore plugging and high surface roughness properties will counteract this gain in hydrophilicity and may counterproductively result in poorer anti-fouling properties. This observation is apparent when comparing data between P25 MMM and neat PVDF membranes. Cao et al. [41] found that the membrane roughness was the most dominant factor on membrane anti-fouling capability under the same operating conditions. Studies have also shown that coarser membranes have higher propensity to absorb impurities from water and possess lower surface energy [52].

For the case of X500 MMM, in which the TiO_2_ nanoparticles’ distribution was enhanced, the HA fouling propensity was significantly reduced, given the lower RFR value when compared to the neat PVDF membrane. The results validated the idea that fine and uniformly distributed TiO_2_ nanoparticles are effective in elevating membrane fouling capability due to improvements in (1) surface smoothness and (2) affinity towards water molecules. These two factors improved water molecules attraction, which promoted the formation of a thin shielding water layer to prevent hydrophobic adsorption of HA macromolecules onto the membrane.

Therefore, after contrasting all the results from different TiO_2_ embedded MMMs, this study reveals that the advantages of nanoparticles embedment can only be realized under the condition when the nanoparticles are uniformly distributed within the polymeric matrix.

#### 3.6.3. Hydraulic Cleaning Properties of Membranes (FRR)

In this study, deposition of HA onto the membranes is considered as reversible membrane fouling as it could be removed by simple hydraulic cleaning [10]. However, when HA molecules are entrapped in pores or adsorbed into the membranes, hydraulic cleaning has limited capability to eliminate or prevent the onset of irreversible membrane fouling. To study the efficacy of hydraulic cleaning on flux recovery of the membranes after cleaning, pure distilled water was recirculated through the system for 4 h at a rate of 0.04 L/min. From the results presented in Figure 6, as expected, permeate flux increases when membranes are washed with pure distilled water. Meanwhile, the permeation flux recovery (represented in terms of FRR) is 82.03% (neat PVDF), 78.58% (PC-20 MMM), 61.89% (P25 MMM), and 78.24% (X500). This observation indicates that flux decline due to pore blocking or surface reversible deposition can be significantly improved using hydraulic force.

#### 3.6.4. Self-Cleaning Ability of Intermittently UV-Irradiated Membranes (IFRR(UV))

Photocatalytic degradation of adsorbed HA onto membranes was performed after physical cleaning by irradiating the membrane surfaces over two cycles with a UV lamp at an intensity of 1.53 mW/cm^2^, where each cycle’s duration is 3 h. After irradiation with UV light, the fouled membrane, which was initially dark brown in colour, gradually turned to light brown and eventually to almost yellowish colour when photocatalytic oxidation occurred. Meanwhile, the colour of the fouled neat PVDF membrane remained the same. After irradiation with UV, the filtration flux of the MMM was effectively enhanced (see Figure 6), evidently showing the positive effect photocatalysis has on TiO_2_ MMMs with enhanced cleaning properties. During photocatalytic degradation, TiO_2_ particles on the MMM surface interact with UV light to generate electrons (e^−^) and holes (h^+^). The photogenerated holes trap and react with H_2_O or O_2_ to yield H^+^ and highly reactive •OH radicals, which is a strong oxidation agent to break down HA molecules by attacking the HA molecules chemically via a hydroxyl addition or hydrogen extraction reaction. Meanwhile, dissolved oxygen (DO) in water can capture electrons and produce unstable O_2_^−^ species that can further react via protonation to yield •OH radicals, which also contribute to the degradation of HA or HA intermediate molecules. Additionally, free radicals are also produced when UV light illumination hydrolyses water molecules on the surface of TiO_2_ nanoparticles and leads to the production of •OH groups.

As photocatalytic cleaning is dependent on the generation of •OH free radicals from water molecules, membranes with stronger hydrophilicity are expected to possess better cleaning capacity under UV irradiation and, by extension, less irreversible fouling. The IFRR(UV) values (0.10 for neat membrane, 5.74 for PC20, 16.56 for P25, 15.30 for X500) clearly indicate that TiO_2_-blended membranes yielded better HA degradation than that of the neat PVDF membrane under UV irradiation. No significant degradation occurred in the absence of TiO_2_, as the UV light alone failed to degrade HA deposited on the neat PVDF membrane surface.

Interestingly, P25 MMM stands out amongst the group by a considerable margin regarding IFRR(UV). This higher photocatalytic activity seen on P25 MMM is attributed to its crystalline properties. P25 consists of 75% anatase, where the Ti ions in tetrahedral coordination are more exposed and more efficiently adsorb water and oxygen in air to produce more hydroxyl groups on the surface of TiO_2_. Meanwhile, the remaining 25% in the rutile phase is capable of stabilizing the photo-excited holes and electron pairs [53], thus slowing down the rate of e^−^ and h^+^ recombination and leading to higher photocatalytic activity.

On the other hand, particle size is another important factor influencing photocatalysis because it directly disturbs the specific surface area of a catalyst (TiO_2_ in this case). The X500 MMM, which has a smaller TiO_2_ particle size (12 times smaller) compared to PC-20 MMM, has more active surface sites and a higher surface charge carrier transfer rate in photocatalysis. Therefore, it is not surprising that X500 MMM also showed excellent photocatalytic activity.

## 4. Conclusions

In this study, HA photodegradation efficiency of different types of TiO_2_-embedded PVDF membranes in a cross-flow membrane filtration system has been demonstrated. PVDF membranes with embedded TiO_2_ saw enhanced hydrophilicity, leading to an increase in pure water permeate flux. Among all PVDF/TiO_2_ MMMs, M3 with homogeneous distribution of X500 nanoparticles displayed the best membrane water permeability (68.17% of membrane water permeability improvement compared to neat PVDF membrane). On the other hand, the PVDF/TiO_2_ MMM’s effectiveness in preventing fouling was found to be primarily dependent on the surface roughness. The degree of TiO_2_ dispersions was shown to be the determinant factor towards the surface pore size, surface free energy, roughness, and HA absorption resistance of a TiO_2_-embedded PVDF membrane. Results showed that fine X500 TiO_2_ nanoparticles with a low tendency of aggregation yielded the lowest fouling tendency (14.69% of RFR), while maintaining a high flux recovery ratio (78.24%). Over a short operation timespan, membrane roughness is found to be a more dominant factor than hydrophilicity in fouling mitigation. The photocatalytic activity of TiO_2_-embeded PVDF membranes towards degradation of absorbed or deposited HA molecules was characterized via UV irradiation on the membranes’ surface. This study affirms the efficacy of UV-induced photocatalytic oxidation towards HA degradation and consequently increased membrane-cleaning efficiency. IFRR(UV) data revealed that P25 TiO_2_, with mixed rutile and anatase crystallinity, produced superior UV-cleaning capacity (16.56% of IFRR(UV)) when dispersed into PVDF membrane, notwithstanding that X500 TiO_2_-embedded PVDF also produced a relatively similar UV-cleaning capacity (15.30% of IFRR(UV)) with advantageously uniform dispersion leading to more active sites and a higher surface charge carrier transfer rate in photolysis. In these regards, X500 MMM containing a well-dispersed TiO_2_ photocatalyst is considered to be an attractive membrane due to its amalgamation of anti-fouling, defouling and UV-cleaning capability.

## Figures and Tables

**Table 1:** Characteristics of TiO_2_ nanopowders.

TiO_2_ Sample	Crystalline Phase	Average Crystalline Size (nm)	Hydrodynamic Cluster Size in Suspension (nm)
PC-20	85% Anatase 15% Rutile	20	461.3
P25	75% Anatase 25% Rutile	~21	200
X500	Anatase	<8	38

**Table 2:** Membrane polymer solution and coagulation bath formulation.

Membrane	PVDF Weight Ratio (%)	DMAc Weight Ratio (%)	Type of TiO_2_	TiO_2_ Concentration (g/L)
M	18	82	-	-
M1	18	82	PC-20	0.01
M2	18	82	P25	0.01
M3	18	82	X500	0.01

**Table 3:** Surface energy properties of contact angle liquids and their viscosities, *η* all at 20 °C [33].

Liquid	*γ^TOT^* (mJ/m^2^)	*γ^LW^* (mJ/m^2^)	*γ^AB^* (mJ/m^2^)	*γ^+^* (mJ/m^2^)	*γ^−^* (mJ/m^2^)	*H* (P or dPa s)
Water	72.80	21.80	51.00	25.50	25.50	0.01000
Glycerol	64.00	34.00	30.00	3.92	57.40	14.90000
Formamide	58.00	39.00	19.00	2.28	39.60	0.04550

**Table 4:** Surface roughness of PVDF/TiO_2_ MMMs and neat PVDF membrane.

Samples	*R_q_* (nm)	Surface Area Ratio (%)
M	30.625	2.1849
M1	55.956	7.7484
M2	107.355	15.1029
M3	29.533	2.3628

Note: the root mean square value of *Z* data (*R_q_*) is the standard deviation of the *Z* values within the given area.

**Table 5:** Static water contact angle, surface tension components, total interfacial tension, and free energy of interaction between membrane surface and water molecules, for the neat PVDF membrane and PVDF/TiO_2_ MMMs.

Membrane	TiO_2_ Type	Contact Angle (°)	*γ_s_^LW^*	*γ_s_^+^*	*γ_s_^−^*	*γ_sw_*	Δ*G_sw_*
M	-	67.56 ± 0.92	50.301	3.812	28.931	3.834	−7.668
M1	PC-20	68.24 ± 0.81	42.656	1.732	26.598	2.664	−5.328
M2	P25	74.44 ± 0.65	44.899	1.628	17.828	10.373	−20.746
M3	X500	71.41 ± 0.76	63.851	5.417	19.763	14.323	−28.646

**Table 6:** Water flux recovery and cleaning properties of neat PVDF membrane and PVDF/TiO_2_ MMMs.

Membrane	Initial Water Flux (L/m^2^ h)	RFR (%)	FRR (%)	IFRR(UV) (%)
M	34.97 ± 2.25	24.24	82.03	0.10
M1	43.21 ± 4.31	23.48	78.58	5.74
M2	37.67 ± 1.11	36.07	61.89	16.56
M3	58.81 ± 1.96	14.69	78.24	15.30
